# Soft error mitigation and recovery of SRAM-based FPGAs using brain-inspired hybrid-grained scrubbing mechanism

**DOI:** 10.3389/fncom.2023.1268374

**Published:** 2023-09-12

**Authors:** Yu Xie, Tingting Qiao, Yizhuang Xie, He Chen

**Affiliations:** Beijing Key Laboratory of Embedded Real-Time Information Processing Technology, School of Information and Electronics, Beijing Institute of Technology, Beijing, China

**Keywords:** brain-inspired, configuration scrubbing, hybrid-grained, single event upset (SEU), SRAM-based FPGA

## Abstract

Soft error has increasingly become a critical concern for SRAM-based field programmable gate arrays (FPGAs), which could corrupt the configuration memory that stores configuration data describing the custom-designed circuit architecture. To mitigate this kind of error, this study proposes a brain-inspired hybrid-grained scrubbing mechanism consisting of fine-grained and coarse-grained scrubbing to mitigate and repair the errors as quickly as possible after an SEU occurrence. Inspired by the human brain's ability to filter out redundant and irrelevant information, we propose a mechanism that can mask invalid position information when errors occur. Compared with the scrubbing of full configuration memory, this mechanism can achieve precise error location and recovery utilizing targeted scrubbing of specific frames or modules. The effectiveness is evaluated by executing fault injection campaigns on the International Symposium on Circuits and Systems 1989 (ISCAS89) benchmark circuits and fault tolerant fast Fourier transform (FT-FFT) circuit. If upsets are detected, they will be repaired with fine-grained or coarse-grained scrubbing depending on their location. The experiment results show that this mechanism can effectively mitigate and repair single-bit upsets (SBUs) and double-bit upsets (DBUs). In addition, the mechanism is proven to be superior in error recovery time and hardware overhead compared to counterpart approaches.

## 1. Introduction

In recent years, “brain-inspired” research has emerged as a significant direction in the fields of artificial intelligence and computer science. Drawing inspiration from the design principles and mechanisms of the human brain's neural system, it offers new insights and approaches for addressing complex computational and processing tasks. In this study, we apply the concept of “brain-inspired” to the design of a hybrid-grained scrubbing mechanism, aiming to enhance fault tolerance and recovery capabilities in SRAM-based FPGAs. By drawing inspiration from the fault tolerance and adaptability of the human brain, we can design systems that are more robust and resilient.

**Figure 1:**
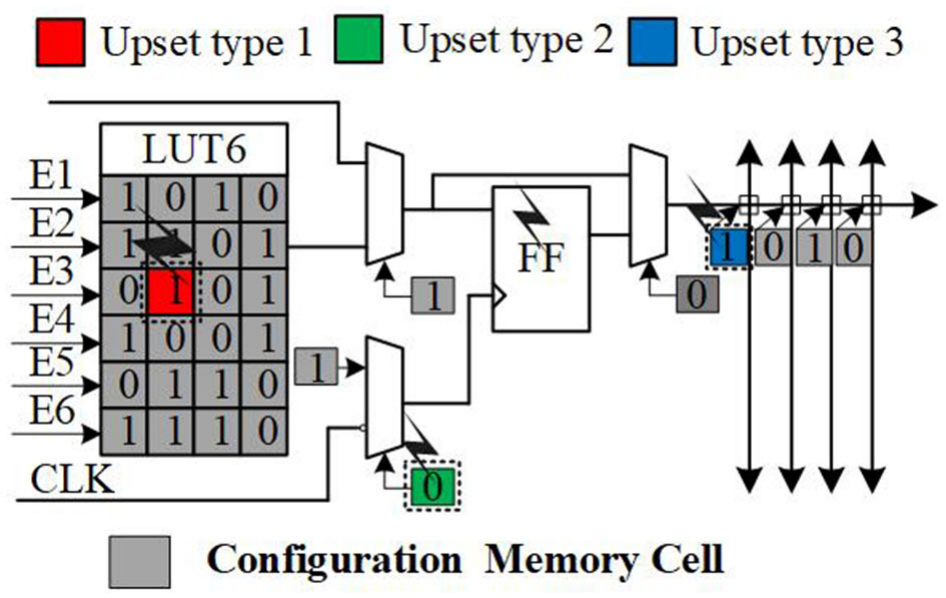
SEUs occurred in the configuration memory of SRAM-based FPGA.

SRAM-based FPGAs have been widely used in security and mission-critical applications (Yang and Fathy, 2009; González et al., 2011; Hartley et al., 2013; Wang et al., 2015), due to their high logic density, low power consumption, reconfiguration feature, and parallel computing capability, especially in aerospace and avionic domains. However, they are extremely sensitive to radiation effects. SRAM-based FPGAs have a number of SRAM cells which are called configuration memory cells. Once the configuration memory cells are hit by energy particles, the configuration bits may be flipped, which is called single event upsets (SEUs) (Karnik and Hazucha, 2004). If SEUs occur in LUT cells or in the cells that control the routing and selectors, it will cause circuit output errors (Morgan et al., 2005), as depicted in Figure 1. These errors have a permanent impact (Nidhin et al., 2017) because these upsets have been latched by the configuration cells. Therefore, certain measures must be taken to prevent the SEUs from affecting the applications and to repair errors.

Recently, many approaches have been proposed to mitigate and repair the errors from different levels (Kastensmidt and Rech, 2016), where there are two different strategies at the design level, one is failure masking and the other is failure recovery.

Failure masking techniques can mask errors through hardware redundancy, such as triple modular redundancy (TMR) (Sterpone and Violante, 2005), partial TMR (Pratt et al., 2008), duplication with compare (DWC) (Johnson et al., 2008), and reduced precision redundancy (RPR) (Pratt et al., 2013). The most classical TMR was first proposed by Von Neumann (1956) and was extensively studied to reduce resource overhead (Samudrala et al., 2004) and improve voter reliability (Kshirsagar and Patrikar, 2009). In addition, algorithm-based fault tolerance (ABFT) (Bosilca et al., 2009) can be used for certain circuits to reduce overhead.

Failure recovery can repair the upsets by dynamically and partially reconfiguring the configuration memory without interrupting the operations (Xilinx UG909, 2019), which is called configuration scrubbing (Herrera-Alzu and Lopez-Vallejo, 2013). Periodic scrubbing is the most basic approach, which periodically reconfigures the configuration memory with the original configuration data. The opposite is readback scrubbing (Michel et al., 2015), which refers to reading back the current bitstream and scanning to find upsets, and reconfiguring the configuration memory with the original bitstream when upsets are detected. Configuration scrubbing can also be classified as internal and external depending on the configuration interface (Berg et al., 2008). External scrubbing uses Select-Map or JTAG (Giordano et al., 2017) to access configuration memory, which typically requires a radiation-hardened device (such as an anti-fuse FPGA, processor, or ASIC) and a radiation-hardened memory (Wang, 2012). Internal scrubbing is typically based on the Internal Configuration Access Port (ICAP) (Guohua et al., 2017), which uses a soft core (HWICAP, 2020) or finite state machine (FSM) (Ebrahim et al., 2012) to control the scrubbing process.

Although the failure masking technique can mask errors, it suffers from large resource overheads and cannot repair upsets, and the main bottleneck of failure recovery is long error detection and recovery time. Thus, we propose a brain-inspired hybrid-grained scrubbing mechanism that combines fine-grained scrubbing and coarse-grained scrubbing.

One key characteristic of “brain-inspired” approaches is their adaptability and fault tolerance. The brain possesses redundant neural networks and neuroplasticity, allowing for reorganization and rewiring in the presence of damaged neurons or connections, thus maintaining fundamental functions and adaptability. By leveraging these features, we have designed a hybrid approach to provide robust error mitigation and recovery capabilities, which can repair single-bit upsets (SBU) and double-bit upsets (DBU) while detecting errors.

Compared with the existing solutions, this approach shows a significant improvement in terms of fault repair rate, recovery time, and area overheads. The main contributions are summarized as follows:

- Soft error mitigation and recovery of SRAM-based FPGAs using a brain-inspired hybrid-grained scrubbing mechanism, which can reduce at least 29.4% error recovery time.
- Analysis of the Xilinx 7-series configuration architecture and calculation of the frame address in fine-grained mechanism.
- A hardware redundancy technology based on ECCs and ABFT to mitigate and mask errors in the coarse-grained mechanism.

The remainder of the study is organized as follows: Section 2 analyzes the Xilinx 7-series configuration architecture and explains how to calculate the frame address. Section 3 presents the brain-inspired hybrid-grained scrubbing mechanism, combining fine-grained and coarse-grained scrubbing mechanisms. In Section 4, the fault injection experiment results are discussed. A comparison with related work is conducted to demonstrate the advantages and validity of this approach. Section 5 concludes the study.

## 2. Methods

### 2.1. Analysis of Xilinx 7-series configuration architecture

**Figure 2:**
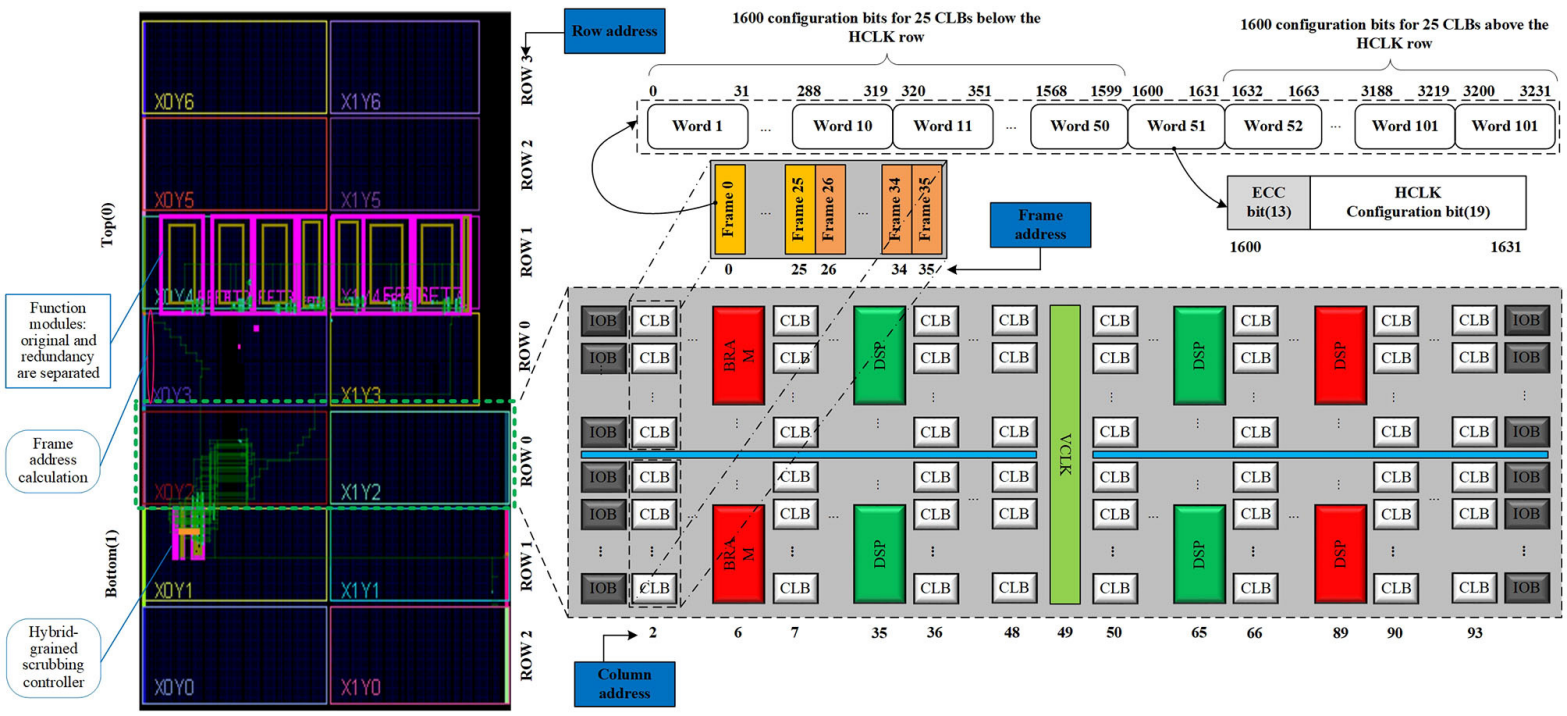
Configuration architecture layout of XC7K325T.

The Xilinx FPGA is composed of a series of function blocks and a set of controls and routes (Xilinx UG470, 2018) as depicted in Figure 2, taking the Kintex-7 XC7K325T (KC705) as a reference. The block types are used to determine certain function blocks, where block 0 is used to define functions of logic, I/O, routing, DSP, etc., block 1 is used for the initial content of the block random access memories (BRAM), and the other types of blocks are used for specific features. The device is physically divided horizontally into two parts: top (0) and bottom (1). Each part is further divided into serval rows depending on the size of the device. There are seven rows in the KC705, the top (0) includes four rows and the bottom (1) includes three rows. This corresponds to the row address. Each row consists of a stack of basic function blocks with a fixed number of columns. Each column is further divided into sub-columns, which are called frames. This corresponds to the minor address.

As can be seen from Table 1, different types of columns have different numbers of frames, depending on the block types.

**Table 1:** Frames per column of the XC7K325T.

**Block**	**Number of frames**
CLB	36
DSP	28
BRAM	28
IOB	42
Clock column	30

### 2.2. Frame address calculation

**Figure 3:**
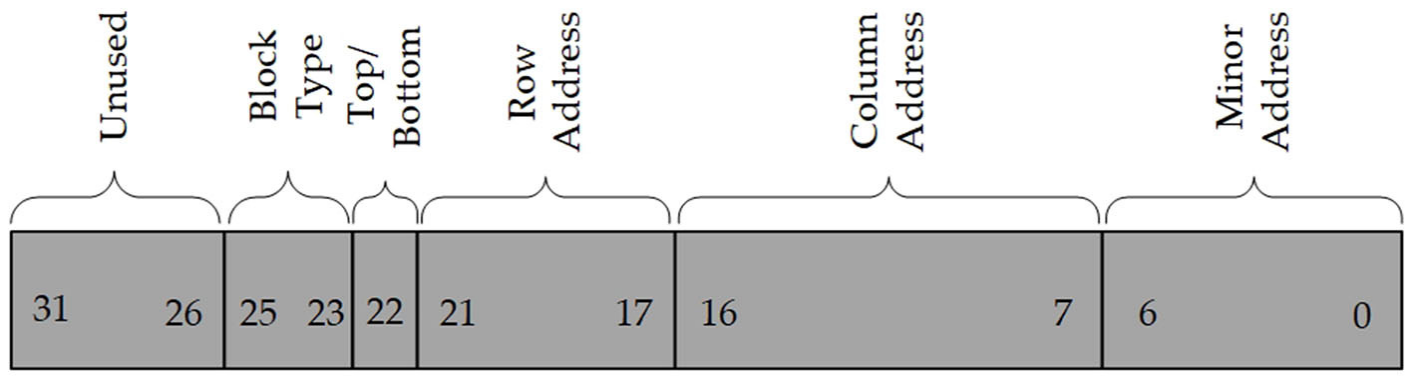
Composition of 32-bit physical frame address.

**Figure 4:**
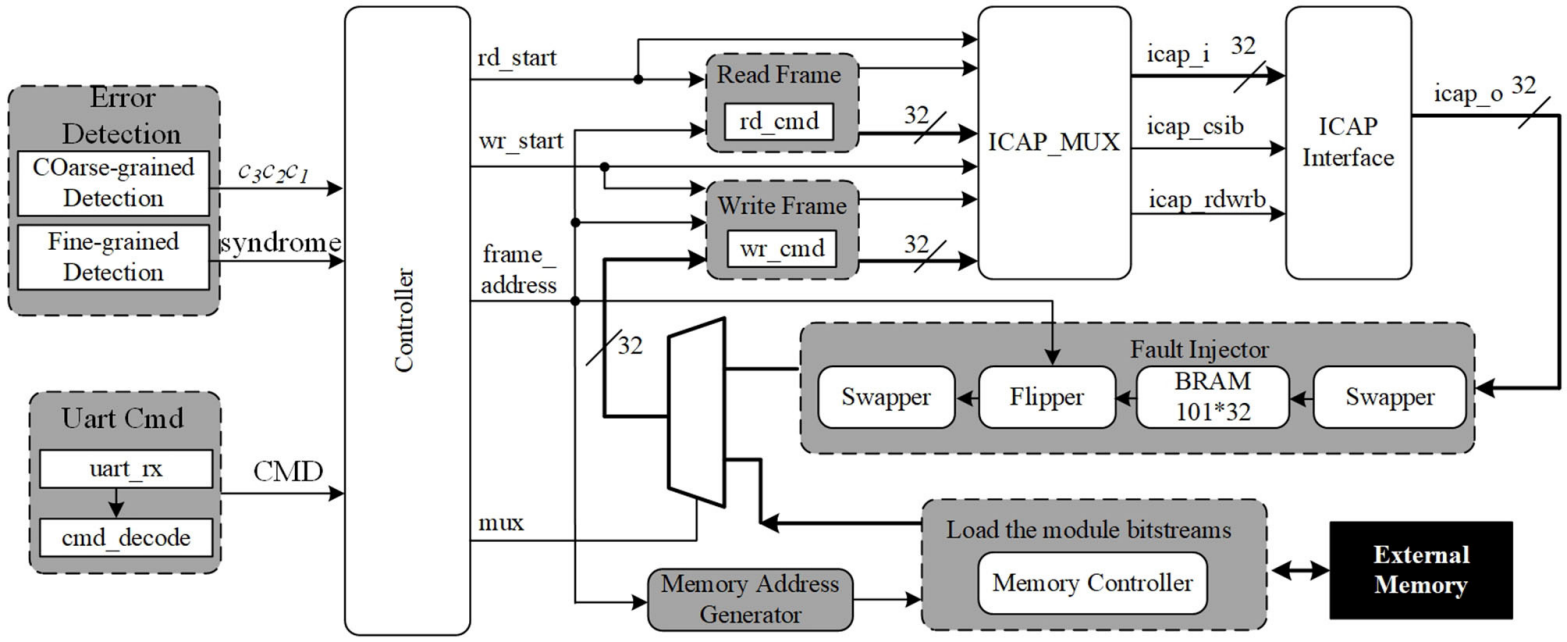
The architecture of brain-inspired hybrid-grained scrubbing mechanism.

The frame is the smallest amount of configuration data that can be read or written. Each configuration frame has a unique 32-bit address that can be divided into 5 parts (Xilinx UG953, 2018), which are block type, top/bottom position, row address, column address, and minor address, as shown in Figure 3. From Section 2.1, we find that the frame address is discontinuous due to block cross-distribution, and different blocks correspond to different frames. We define these non-contiguous frame addresses as physical frame addresses (PFAs), and the addresses obtained by sequentially arranging PFAs are called linear frame addresses (LFAs). The function of an SRAM-based FPGA is determined by configuration data called bitstream, and the bitstream consists of frame data. Since Xilinx does not disclose the relationship between frame data and PFA in a bitstream, it is difficult to reconfigure the error frame with a correct frame to repair upsets. We derived this relationship by analyzing the configuration architecture and bitstream. For example, if a module is placed in top (0), row 0, column 2, as shown in Figure 4, then its PFA is 00000100 to 00000123(h), and the corresponding LFA is 72 to 107(d). Therefore, the corresponding frame data of the module in the bitstream is the 72nd to 107th frame.

## 3. Mechanism

### 3.1. Brain-inspired hybrid-grained scrubbing mechanism

The architecture of the brain-inspired hybrid-grained scrubbing mechanism is shown in Figure 4. It mainly consists of an error detection module, scrubbing controller, fault injector, Internal Configuration Access Port (ICAP), universal asynchronous receiver/transmitter (UART), and external memory.

#### 3.1.1. Error detection module

The detection logic of fine-grained and coarse-grained mechanisms are implemented in it, and the error detection signals will be input into the scrubbing controller.

#### 3.1.2. Scrubbing controller

It is implemented by Verilog and used to control the read frame and write frame, which are used to read frames from or write frames to the configuration memory.

By utilizing the ICAP module, a fault injector can be implemented. The fault injection or error repair is a simple read–modify–write process. When we finish the procedure of injecting fault, the FPGA will operate the data that include errors. Then, the brain-inspired hybrid-grained scrubbing will come into force. ICAP is a configuration interface that allows to access configuration memory by FSM or an embedded processor. External memory is used to store the original configuration data. The UART is used to interact with the scrubbing controller with a specific command format.

**Figure 5:**
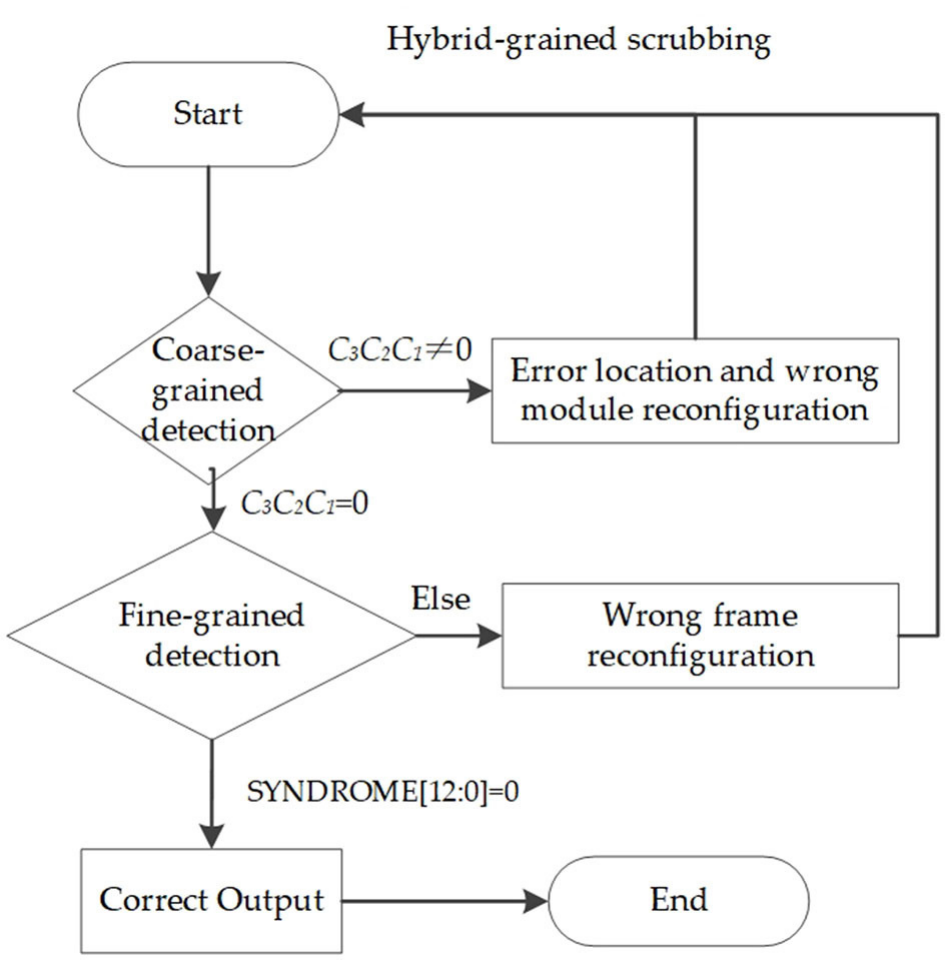
The recovery flow of brain-inspired hybrid-grained scrubbing.

The coarse-grained scrubbing mechanism can repair SBU and DBU with a shorter repair latency compared with the fine-grained mechanism; this is applied for critical designs. Only the upsets that cause the output error can be detected and repaired in the coarse-grained mechanism, the others will accumulate in configuration memory, which can be repaired by the fine-grained mechanism. Thus, this study combines fine-grained and coarse-grained scrubbing mechanisms to provide the strongest mitigation and recovery capabilities. We enable both mechanisms at the same time. If errors occur in FT-FFT modules, the upsets will be detected and repaired by the coarse-grained mechanism, and if it is not, they will be detected and repaired by the fine-grained mechanism. Figure 5 shows the entire recovery flow of brain-inspired hybrid-grained scrubbing when SBU or MBU is injected.

As shown in Table 2, this approach occupies 385 LUTs, 56 FFs, and 1.5 RAMs, <1% of the total resources in KC705. Compared to the soft error mitigation (SEM) IP (Le, 2012) created by Xilinx, this approach has fewer hardware overheads and therefore has fewer sensitive areas for higher reliability.

**Table 2:** Hardware resource comparison.

	**Hardware overhead**			**I/O**
	**LUTs**	**FFs**	**BRAM (RAM36)**	
This approach	385	56	1.5	1
SEM	559	139	1.5	56

The fine-grained scrubbing mechanism enables precise localization and repair of errors such as single-bit upsets, double-bit upsets, and multiple-bit upsets. On the other hand, the coarse-grained scrubbing mechanism employs hardware redundancy techniques and error detection and correction codes to mask and repair errors by reconstructing faulty modules. The integration of these two mechanisms has led to significant improvements in terms of error repair rate, recovery time, and resource utilization.

### 3.2. Fine-grained scrubbing mechanism

**Figure 6:**
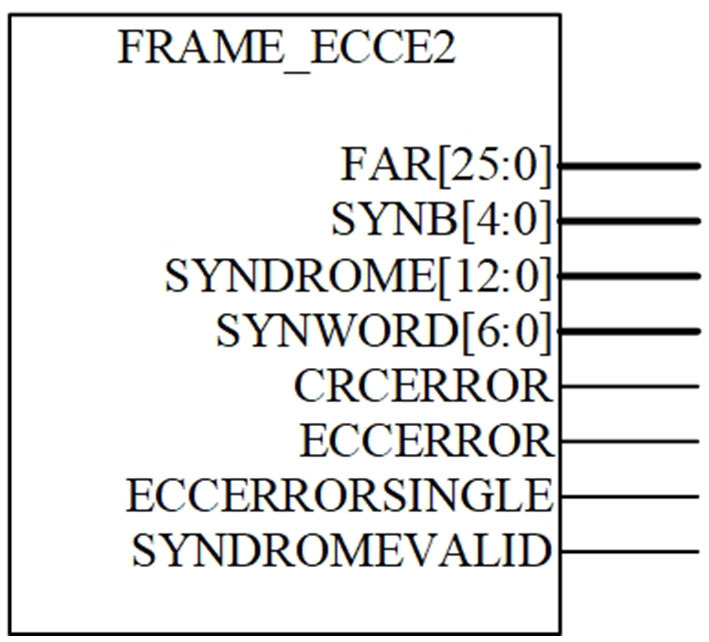
Diagram of FRAME_ECCE2.

The fine-grained scrubbing mechanism utilizes two built-in features of Xilinx, which are the readback cyclic redundancy check (CRC) circuit and FRAME_ECCE2 primitive. Readback CRC is a dedicated circuit to continuously read back the bitstream in the background while computing the global CRC value. At the end of the readback, if the computed CRC does not match the golden CRC, it indicates that a CRC error has occurred. FRAME_ECCE2 is a primitive consisting of a 12-bit standard Hamming code and an extra parity bit, which allows to detect and correct a single error and detect (but not correct) a double error (SEC/DED). Its block diagram is shown in Figure 6.

Each configuration frame in Xilinx FPGAs contains 101 words, of which the 13-bit extended Hamming is stored in the 51st word, as depicted in Figure 3. To start using the FRAME_ECCE2 circuit, it is necessary to first invoke the Readback CRC circuit to read back the current bitstream. Then, FRAME_ECCE2 utilizes the bitstream to compute the SYNDROME [12:0]. If SYNDROME [12:0] is not equal to zero, it indicates that the current frame has an error and FAR [25:0] will give the PFA of the current frame. If the odd numbers of bits are flipped, the parity will be incorrect, resulting in the SYNDROME [12:0] being incorrect. Therefore, the FRAME_ECCE2 always detects double and all odd-number bit upset in a frame, while for even-number upset >2, it is not always detectable. If FRAME_ECC does not detect an even-number upset >2, then it will eventually be detected by the CRCERROR signal at the end of the readback.

**Figure 7:**
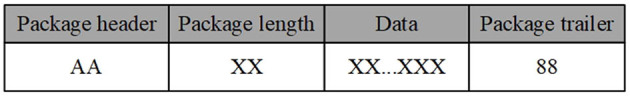
The format of fault injection command.

The fine-grained mechanism consists of a readback CRC circuit and FRAME_ECCE2 primitive, a scrubbing controller, ICAP, UART, and an external memory. Readback CRC and FRAME_ECCE2 are responsible for detecting upsets. ICAP is a configuration interface that allows to access configuration memory by FSM or an embedded processor. External memory is used to store the original configuration data. The scrubbing controller implemented by Verilog is used to control the read frame and write frame, which are used to read frames from or write frames to the configuration memory. The UART is used to interact with the scrubbing controller in the format of a specific command, as shown in Figure 7.

- Command to enter idle state: AA084988.
- Command to inject error with PFA: AA28XXXXXXXXXX88.
- Command to enter Observation state: AA084F88.

The UART receives three types of commands above. In the idle state, the scrubbing controller receives the fault injection command and the command to enter the observation state. To evaluate the effectiveness of the brain-inspired hybrid-grained scrubbing mechanism, we implemented a fault injector by reading–modifying–writing a frame, which can simulate SBUs or MBUs. In the observation state, the system automatically detects and repairs upsets. If upsets are detected by the SYNDROME [12:0] signal, it is repaired by reconfiguring the error frame with the correct frame. If it is detected by the CRCERROR signal at the end of the readback, since the address of the fault frame is unknown, the scrubbing controller will reconfigure the whole configuration memory to repair the error.

**Figure 8:**
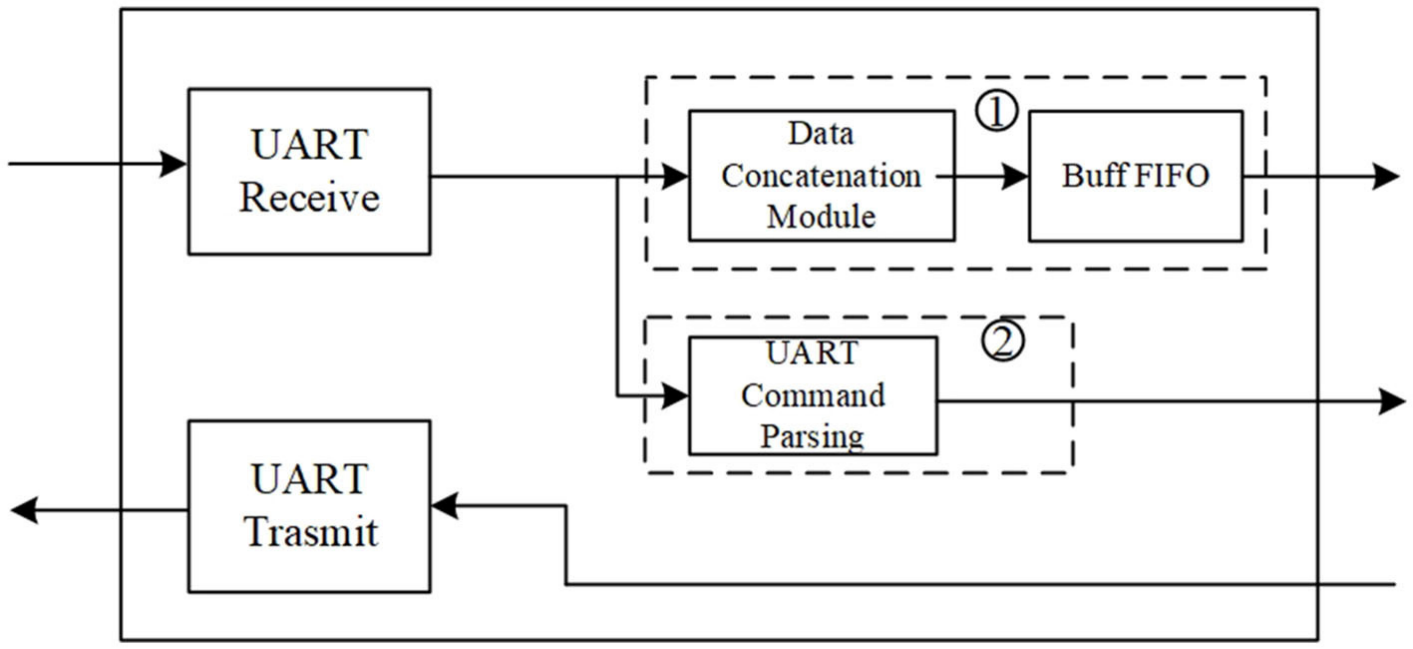
Whole circuit structure of the fault injection command module.

The whole circuit structure of the fault injection command module (shown in Figure 4) is shown in Figure 8, consisting of the UART receive module, UART transmit module, data concatenation module, buffer FIFO, and fault injection command parsing module. Line ① is responsible for delivering the configuration data corresponding to the .rbd file, .msd file, and FFT modules to the DDR read/write control module, then writes the data to the external DDR3 memory. Line ② is responsible for receiving control information sent from the host computer and parsing it into corresponding control commands, facilitating interaction between the host computer and the brain-inspired hybrid-grained scrubbing controller.

(1) UART receive and transmit module:

This module consists of UART receive and transmit circuits and operates with a data format of 1 start bit, 8 data bits, 1 stop bit, no parity bit, and a baud rate of 115,200 bps.

(2) Data concatenation module:

The data concatenation module converts the .rbd file, .msd file, and configuration data of the FFT backup module from an 8-bit data width to a 512-bit data width.

(3) Buffer first input first output (FIFO):

The buffer FIFO module is composed of a 512-bit wide and 512-bit deep FIFO. Its main function is to buffer the data received from the data concatenation module, facilitating read access by the DDR read/write control module.

(4) UART command parsing module:

The UART command parsing module decodes each frame of control information received via the UART. It extracts the control commands: Enter Idle State Command, Error Injection Command, and Observation Command. The command parsing is implemented using a state machine, consisting of four states: Frame Header Parsing, Frame Length Parsing, Frame Data Reception, and Frame Footer Parsing. The state machine starts in the Frame Header Parsing state and transitions to the Frame Length Parsing state upon detecting the frame header data (0xAA). If the received frame length matches the expected values (0x08, 0x10, 0x20, or 0x28), it moves to the corresponding Frame Data Reception state; otherwise, it returns to the Frame Header Parsing state. In the Frame Data Reception state, after receiving the frame data of the expected length, it transitions to the corresponding Frame Footer Parsing state. If the frame data length does not match the expected length, it directly returns to the Frame Header Parsing state. If the frame footer is 0x88, the parsing is successful, and the command is considered valid. If the frame footer is not 0x88, the frame data is discarded, and it returns to the Frame Header Parsing state. After completing the frame data parsing, it automatically returns to the Frame Header Parsing state.

For the Error Injection Command, the data information corresponds to the address for fault injection, consisting of 40 bits. The bits 35–39 are all “0”, bit 34 represents the quadrant address, bits 29–33 represent the row address, bits 19–28 represent the column address, bits 12–18 represent the sub-address, bits 5–11 represent the word address (which word within the frame), and bits 0–4 represent the bit address (which bit within the word).

The ports of the UART communication and command parsing module are shown in Table 3. After power-on, line 1 is connected while line 2 is disconnected. The UART receive module receives the .rbd file, .msd file, and configuration data corresponding to the FFT backup module from the host computer. The received data are processed by the data concatenation module to generate 512-bit data, and if the buffer FIFO is not full, the data are written into the buffer FIFO. At the same time, when the DDR read/write control module detects that the buffer FIFO is not empty, it reads the data and writes it to the specified address in the external DDR3 memory. Once the .rbd file, .msd file, and configuration data for the FFT backup module are written into the DDR3, line 1 is disconnected and line 2 is connected, maintaining this state. When line 2 receives control information, it parses the data packet and generates commands for the main controller. The UART transmit module is used to send the current status of the fault injection, fault detection, and repair of the hybrid-grained scrubbing controller back to the host computer.

**Table 3:** The ports of the UART communication and command parsing module.

**Signal name**	**Bit width**	**Direction**	**Description**
Clk	1	Input	System clock
rst_n	1	Input	System reset
i_Rx_Serial	1	Input	UART receive
rd_en	1	Input	Enable read buffer FIFO
sel_0	1	Input	Switch between line ① and ,② “0” for line ①, “1” for line②
fifo_empty	1	Output	Buffer FIFO empty signal
data	512	Output	Buffer FIFO output
inj_en	1	Output	Enable fault injection
inj_addr	40	Output	Fault injection address
idle_en	1	Output	Enter idle state command
readback_en	1	Output	Enable readback data command
readback_addr	32	Output	Readback data frame address
scan_en	1	Output	Readback data frame address

### 3.3. Coarse-grained scrubbing mechanism

Long error detection and recovery time and the inability to mask errors are two major drawbacks of fine-grained scrubbing mechanisms. With the KC705 as a reference, it takes 23.5 ms to scan the entire configuration memory. On average, it will take half of the configuration scan time to find an error (11.25 ms). This is unacceptable for high-reliability and high-efficiency applications. Therefore, it is necessary to introduce a mechanism to repair errors more quickly than fine-grained scrubbing while detecting errors.

Actually, both failure masking and scrubbing techniques are used in coarse-grained mechanisms. This study utilizes hardware redundancy to mask errors in coarse-grained, including redundancy with ECCs and ABFT (Gao et al., 2015). If an error is detected in a certain module, the error will be masked and a reconfiguration will be performed to repair the failed module. With Xilinx floor planning technology, we limit the original and redundant modules to a specific position. According to the analysis in Section 2, we can calculate the LFA of each module. Then, we can write a script to extract the configuration frames data of the corresponding module from the bitstream and store them in the external memory. Once upsets occur in a certain module, it will be detected immediately and the accurate frame address of this module will be located because this approach does not need to read back bitstream to check for upsets frame-by-frame. If any one of the modules fails (including the redundancy module), the other modules can mask the errors and output, while the controller executes a reconfiguration to repair the failed module. The greatest advantage of coarse-grained scrubbing is the reduction of recovery time because we do not need to check for upsets frame-by-frame in the function modules (which often occupy major memory configuration of the FPGA).

**Figure 9:**
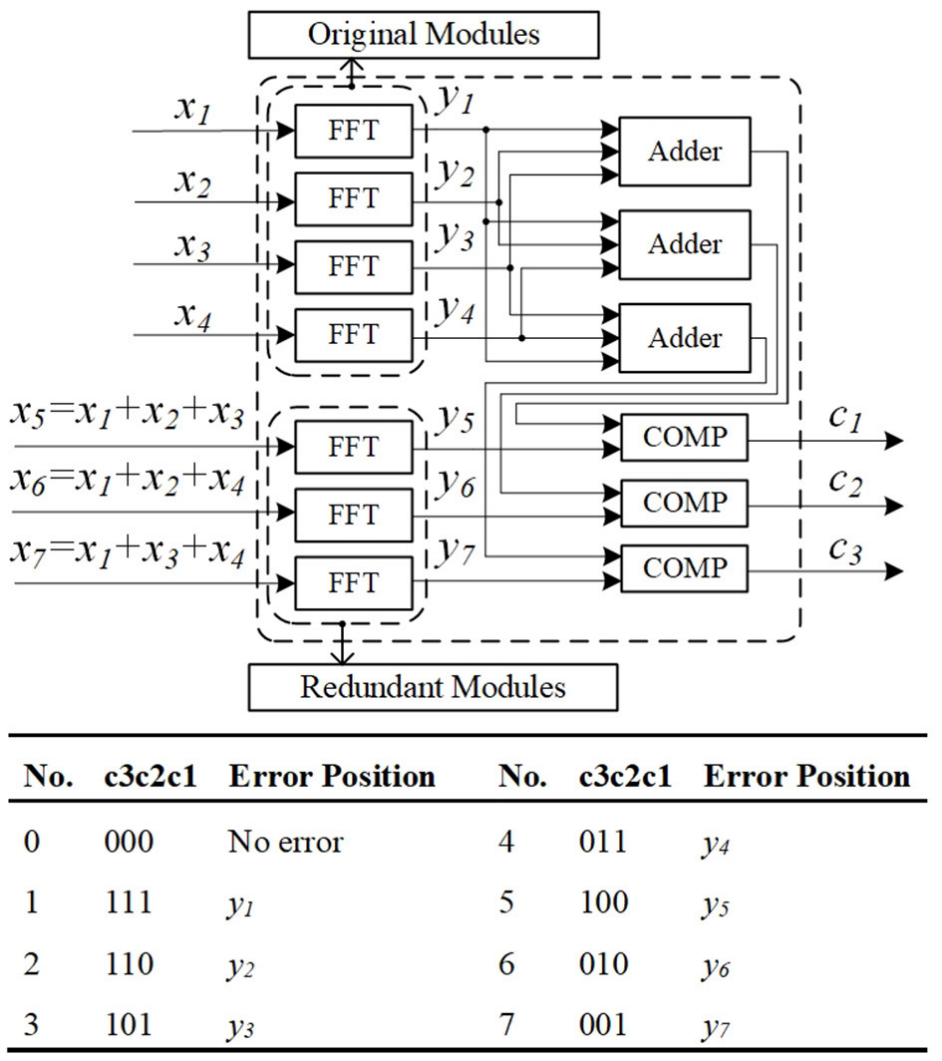
Coarse-grained mechanism error detection scheme.

The error detection scheme of the coarse-grained mechanism is shown in Figure 9, taking fast Fourier transform (FFT) as an example. Traditional TMR for FFT needs large extra overhead (twice more than that used in the original design), while the fault-tolerant FFT (FT-FFT) design can lower the cost to about 1.75 (7/4). Hence, we can save 41.7% (1–1.75/3) hardware overhead compared to TMR.

The main principle of this approach is that for any linear operation, the output of a linear combination of multiple inputs is equal to the same linear combination of their individual outputs, an example is expressed in Equation (1).


(1) $$\{\begin{array}{c} y_{5}(c_{1})=y_{1}+y_{2}+y_{3}\\ y_{6}(c_{2})=y_{1}+y_{2}+y_{4}\\ y_{7}(c_{3})=y_{1}+y_{3}+y_{4} \end{array}$$

Generally, we consider that only one error will occur in several modules at the same time. Assume that the check bits are *c*_3_*c*_2_*c*_1_, which is determined by Equation (1). Different error patterns are summarized as shown in Figure 9. If an expression in Equation (1) does not hold, the corresponding check bit will be set to 1. For example, if *y*_3_ is wrong, then the *c*_3_*c*_2_*c*_1_ will be set to 101 and the output can be reconstructed by Equation (2), therefore, the method is not unique.


(2) $$\begin{array}{l} y_{3}=y_{5}-y_{1}-y_{2} \end{array}$$

## 4. Fault injection experiments

The experiments are carried out in KC705 Xilinx FPGA and all circuits are run at 100 Mhz. We first evaluated the effectiveness of the fine-grained and coarse-grained mechanisms by randomly injecting faults into the ISCAS89 circuit and the FT-FFT circuit, respectively. Then, we enabled both mechanisms at the same time and evaluated the brain-inspired hybrid-grained scrubbing mechanism by injection faults into the FT-FFT circuit.

The error recovery time (*T*_*R*_) and the error repair rate (μ) are two important evaluation criteria. *T*_*R*_ is defined as the sum of the error detection time (*T*_*D*_) and error correction time (*T*_*C*_), as expressed in Equation (3).


(3) $$\begin{array}{l} T_{R}=T_{D}+T_{C} \end{array}$$

In Xilinx FPGAs, essential bits are defined here as those bits associated with the circuitry of the design and are a subset of the device configuration bits. Critical bits are defined here as those bits that cause a functional failure. In this study, μ is defined as the number of upsets corrected (*N*_*C*_) divided by the number of total upsets that can be injected (*N*_*T*_), as expressed in Equation (4). *N*_*T*_ depends on the number of essential bits of KC705. In addition, the number of upsets that caused circuit output error is represented by *N*_*E*_, which depends on the number of critical bits.


(4) $$\begin{array}{l} \mu =N_{C}/N_{T} \end{array}$$

### 4.1. Error recovery time

The comparison of *T*_*R*_ is shown in Table 4. Soft Error Mitigation Controller LogiCORE IP (SEM IP) (Xilinx PG036, 2015) is an IP created by Xilinx to solve soft errors. It has three modes, and here we only compare the frame replacement mode. Stoddard et al. (2016) proposed a scrubbing method, which combines internal and external scrubbing. We have conducted statistical analysis on the average fault recovery time of the brain-inspired hybrid-grained scrubbing mechanism.

**Table 4:** Results of error recovery time.

**Error recovery time (ms)**		
	**SBU**	**DBU**
Fine-grained	11.7541	11.7541
Coarse-grained	0.3666	0.3666
Hybrid-grained (average)	8.298	8.298
SEM IP (Xilinx PG036, 2015)	12.58	12.58
Stoddard et al. (2016)	11.7545	13.61

This study adopts two different scrubbing mechanisms to refresh and repair the FFT circuit area and the non-FFT circuit area separately. As the fine-grained and coarse-grained scrubbing mechanisms govern different areas, it is necessary to calculate the weighted average of the error recovery time (*T*_*R*_) for both mechanisms based on their respective coverage areas.

Let *N*_*fine*−*grained*_ represent the total number of configuration frames managed by the fine-grained scrubbing mechanism responsible for detection and repair, and *N*_*coarse*−*grained*_ represent the total number of configuration frames managed by the coarse-grained scrubbing mechanism. The average fault recovery time for the hybrid-grained, fine-grained, and coarse-grained mechanisms is denoted as _*T*_*R*_*hybrid*−*grained*_, _*T*_*R*_*fined*−*grained*_, _*T*_*R*_*coarse*−*grained*_, respectively. The weighted average fault recovery time for the hybrid-grained mechanism _*T*_*R*_*hybrid*−*grained*_ can be obtained by Equation (5) as follows:


(5) $$\begin{array}{l} {T_{R}}_{hybrid-grained}=\frac{\left(N_{fine-grained}\times {T_{R}}_{fine-grained}+N_{coarse-grained}\times {T_{R}}_{coarse-grained}\right)}{\left(N_{fine-grained}+N_{coarse-grained}\right)} \end{array}$$

Considering the differences in the coverage areas managed by the fine-grained and coarse-grained scrubbing mechanisms, this weighted average computation allows for a comprehensive evaluation of the hybrid-grained approach's overall performance in terms of fault recovery time.

As can be seen from Table 4, this approach has the smallest *T*_*R*_, so we can repair errors in the shortest time. The unit of *T*_*R*_ is milliseconds. Compared with using fine-grained scrubbing alone, the proposed hybrid-grained method achieves a further reduction of 29.4% in fault recovery time.

### 4.2. Fault injection and scrubbing experimental results

Table 5 shows the faults injection results, where 10,000 upsets for SBU and DBU have been randomly injected into the configuration memory for some ISCAS89 benchmark circuits, coarse-grained fault tolerant FFT circuits, and brain-inspired hybrid-grained circuits. It can be seen from the *N*_*E*_ columns that not all upsets lead to wrong circuit output, and bits corresponding to these upset positions can be considered as non-critical bits. This is also foreseeable because not all configuration bits are directly related to the circuit's output. The results indicate that both the fine-grained mechanism and coarse-grained mechanism can detect and correct SBU or DBU, with an error repair rate μ of 100%.

**Table 5:** Results of fault injection and scrubbing experiments.

**Fault injection and scrubbing experimental results (numbers)**								
	**SBU**				**DBU**			
**Fine-grained ISCAS89 benchmark circuits**	***N**_*E*_*	***N**_*C*_*	***N**_*T*_*	μ	***N**_*E*_*	***N**_*C*_*	***N**_*T*_*	μ
S510	1,121	10,000	10,000	100%	1,457	10,000	10,000	100%
S713	1,605	10,000	10,000	100%	2,090	10,000	10,000	100%
S820	2,319	10,000	10,000	100%	3,000	10,000	10,000	100%
S832	2,878	10,000	10,000	100%	3,576	10,000	10,000	100%
S953	3,517	10,000	10,000	100%	4,345	10,000	10,000	100%
S1196	4,110	10,000	10,000	100%	5,005	10,000	10,000	100%
S1238	4,662	10,000	10,000	100%	5,715	10,000	10,000	100%
S1494	5,356	10,000	10,000	100%	6,346	10,000	10,000	100%
S5378	5,916	10,000	10,000	100%	7,115	10,000	10,000	100%
S9234	7,120	10,000	10,000	100%	8,657	10,000	10,000	100%
Coarse-grained FT-FFT circuit	2,980	2,980	2,980	100%	3,765	3,765	3,765	100%
Hybrid-grained circuit	3,035	3,035 + 6,965	10,000	100%	3,876	3,876 + 6,124	10,000	100%

The effectiveness of the coarse-grained scrubbing mechanism is evaluated by randomly injecting 10,000 SBU and DBU in redundant and original FFT modules of FT-FFT circuits. Since the coarse-grained mechanism does not read back the bitstream and check it for upsets frame-by-frame, but directly compares the output results to immediately find the module with errors, the *T*_*D*_ can be ignored. *T*_*C*_ depends on the number of frames of the FFT module. The coarse-grained mechanism has an order of magnitude improvement in *T*_*R*_ compared to the fine-grained mechanism and other solutions. It should be noted that the coarse-grained scrubbing mechanism can only be used for fault recovery of critical bits upsets, which corresponds to the upsets that affect the FT-FFT output results in this experiment.

To evaluate the effectiveness of the brain-inspired hybrid-grained scrubbing mechanism, we enable both fine-grained and coarse-grained scrubbing mechanisms at the same time. Similarly, we randomly inject 10,000 SBU and DBU into the FT-FFT circuit and record the results of the experiment. The fault injection results are shown in the last row of Table 5. The upsets that cause the circuit's output error are repaired by the coarse-grained scrubbing mechanism, and the rest are repaired by the fine-grained scrubbing mechanism. Experimental results show that combining these two mechanisms can provide the strongest error mitigation and recovery capabilities with = 100%.

### 4.3. Tests for SAR imaging application

**Figure 10:**
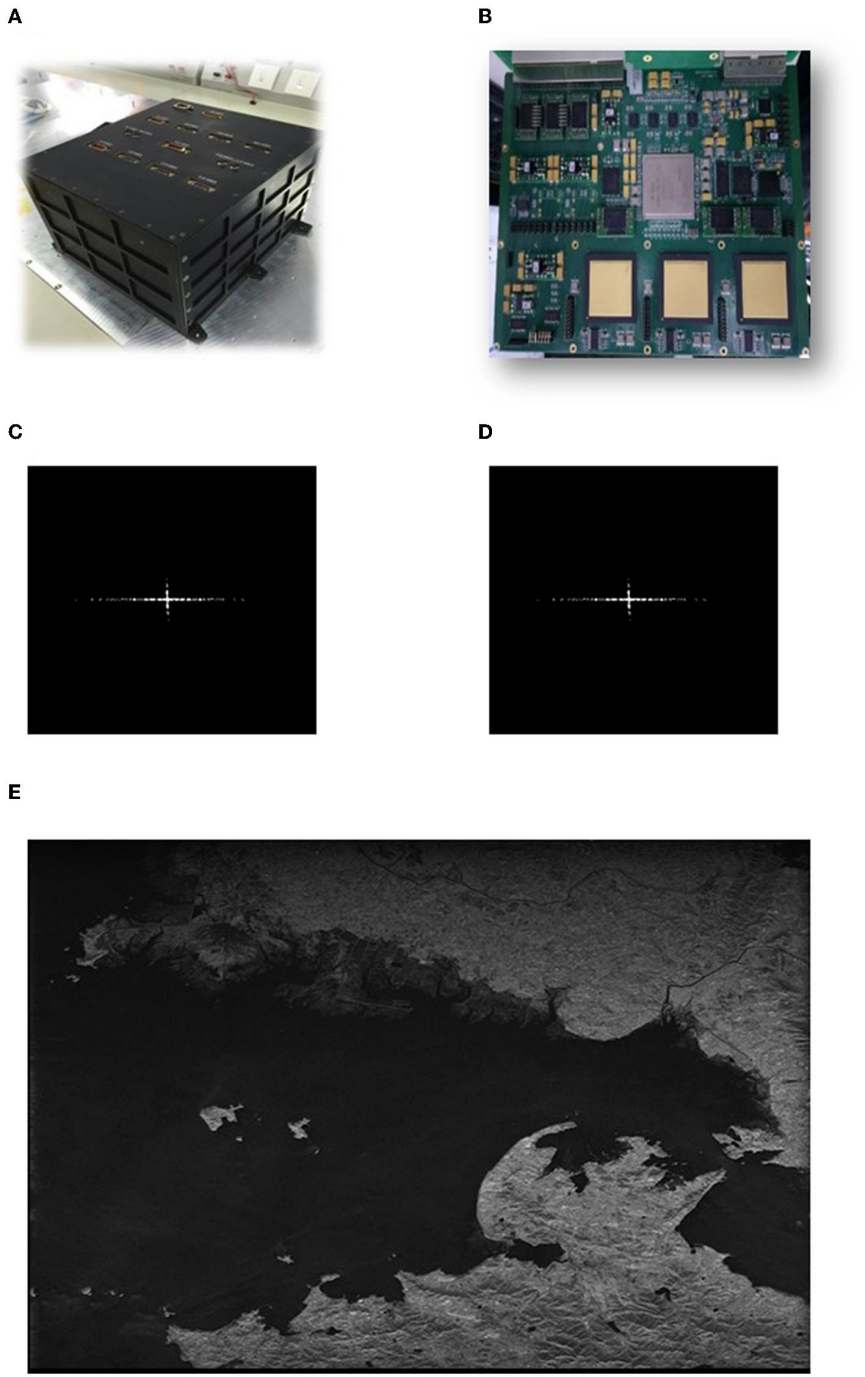
Test results for SAR imaging application. **(A)** Prototype of the system. **(B)** The processing board. **(C)** Original point target result. **(D)** Average result with 1,000 fault injection tests. **(E)** Actual scene imaging results with this mechanism.

Considering the practical application, the soft error mitigation and recovery performance should be tested further. To make sure whether the proposed mechanism is valid for SAR imaging, we use a 16K^*^16K Chirp-Scaling SAR imaging system developed by this laboratory (Beijing Key Laboratory of Embedded Real-Time Information Processing Technology, Beijing Institute of Technology) for the tests. The prototype of the system is shown in Figure 10A and the processing board is shown in Figure 10B. We apply this brain-inspired hybrid-grained scrubbing mechanism to the processing board to improve its fault tolerance.

We adopt a chirp scaling (CS) imaging algorithm for the system-level SAR verification tests. The calculations of FFT/IFFT of the processing flow are protected by this brain-inspired hybrid-grained scrubbing mechanism. A typical SAR system test scenario is a point target scene. We use the fault injection command in a fine-grained mechanism to mimic errors and repeat 1,000 tests, the average imaging results are shown in Figures 10C, D. From the comparison of the results with the original result, we can see that the proposed mechanism can achieve good protection on FFT modules in SAR imaging. In addition, we apply this mechanism on an actual scene imaging system, results shown in Figure 10E, and verify the validity and availability of this design.

## 5. Conclusion

This study proposed a brain-inspired hybrid-grained scrubbing mechanism, combining fine-grained and coarse-grained mechanisms that can mitigate and repair SBU or DBU. In summary, the application of “brain-inspired” principles provides an innovative design approach for this hybrid-grained scrubbing mechanism, offering new perspectives for enhancing reliability and performance in SRAM-based FPGAs. By combining the principles and techniques inspired by the brain, we can better distinguish error location and interferences, leading to more reliable and efficient soft error mitigation and recovery capabilities for SRAM-FPGA. In fine-grained mechanism, we analyze the configuration architecture and bitstream to resolve the problem of correspondence between the PFA and bitstream, so that faults can be repaired by run-time dynamic partial reconfiguration of frame or module. This mechanism experiences a significant reduction of *T*_*R*_ compared to full reconfiguration. We utilize Xilinx's built-in features to detect errors, which not only simplifies the circuit structure but also provides higher reliability. This approach accounts for less resources than SEM IP. In the coarse-grained mechanism, we use a hardware redundancy technology based on ECCs and ABFT to mitigate and mask errors, saving 41.7% overhead compared to TMR. The proposed approach was evaluated through fault injection, and the results demonstrated that this mechanism can mitigate and repair upsets with a repair rate of 100%. At last, this brain-inspired hybrid-grained mechanism can reduce at least 29.4% of the time of *T*_*R*_ compared to counterpart approaches.

There exists a notable limitation that the current implementation is primarily applicable to FPGA architectures, relying on the linear frame addresses of FPGA configuration files for refresh and reconstruction. To further enhance the effectiveness and versatility of this approach, future work should focus on extending its application to the general circuit domain. By overcoming the dependency on FPGA-specific configuration features, the method can be adapted to other types of circuits, including ASICs and custom-designed integrated circuits. This extension would open up new opportunities for improving reliability and performance in a broader range of electronic systems, addressing soft error mitigation and recovery challenges beyond the realm of FPGAs. With continued research and development, the brain-inspired principles integrated with fine-grained and coarse-grained techniques hold the potential to revolutionize fault-tolerance strategies and make electronic systems more robust and reliable across various applications.

## Data availability statement

The original contributions presented in the study are included in the article/supplementary material, further inquiries can be directed to the corresponding author.

## Author contributions

YuX: Conceptualization, Formal analysis, Writing—original draft. TQ: Software, Validation, Writing—review and editing. YiX: Software, Validation, Writing—review and editing. HC: Conceptualization, Methodology, Writing—review and editing.
